# Community case studies: an interpretative phenomenological analysis on sexual abuse in urban Chennai

**DOI:** 10.3389/fpsyg.2024.1352066

**Published:** 2024-03-14

**Authors:** Niranjana Ganesan, Bhuvaneswari Gopalakrishnan

**Affiliations:** School of Social Sciences and Languages, Vellore Institute of Technology, Chennai, India

**Keywords:** sexual abuse, phenomenology, IPA, lived experiences, pandemic, COVID-19, pain, teenage

## Abstract

This study attempts to explore the lived experiences of sexual abuse during COVID-19 in a big metropolitan city in India, with a special interest in understanding the contemporary problems faced by teenagers. Any Phenomenological enquiry begins with identifying and determining the suitability of the participants. In this case, the participants are teenage girls and boys, who have experienced one or other forms of sexual abuse during the COVID-19 pandemic. By restricting the act of sexual abuse that happened during a pandemic, this research brings attention to the medium (material), social conditions, and the role of the cultural world in the act of sexual abuse. It begins by distributing a questionnaire to 500 participants to identify the participants who had experienced one or other forms of sexual abuse during the aforementioned time. Secondly, brief interviews with the identified participants are conducted to record their lived experience of sexual abuse. Using this collected narrated experience as a reservoir for phenomenological reflection, this research aims to uncover moments of lived experience emphasizing spatial, corporeal, and temporal aspects as well as provide greater depth in understanding sexual abuse in the context of teenage lives. It helps to understand the different forms of sexual abuse experienced by teenagers as well as portrays how space plays a major role in the act. This article aims to highlight the role of the material world in the act as well as how the social, political, and cultural contexts are materialized in the act of sexual abuse. Also, this article analyses how the existing intervention mechanisms support the prevention of sexual abuse in different social settings as well as emphasizes the gaps in the intervention mechanisms apropos the chosen narrative data. To sum up, this study aims to create awareness, provides prevention mechanisms considering the role of the material world, and advocates for SDG 16 (Promote justice, peaceful and inclusive societies) and SDG 5 (Achieve gender equality and empower all women and girls).

## Introduction

Sexual abuse refers to any sexual activity perpetrated without consent. It includes unwanted touching, forced sexual activity, oral sex, and rape among other sexual acts. WHO^1^ defines sexual abuse as *Actual or threatened physical intrusion of a sexual nature, whether by force or under unequal or coercive conditions*. Sexual abuse becomes harassment once the position/power comes into the picture. UN’s Sustainable Development Goal of promoting justice, peaceful and inclusive societies, and gender equality emphasizes any form of discrimination and violence against people.^2^ Henkhaus’s (2022) study on childhood sexual abuse and its impact on human capital and economic wellbeing brings attention to the problem of poor educational attainments and a decrease in the labor market outcomes. This foregrounds sexual abuse as a public health crisis and alerts us to the importance of robust prevention mechanisms. A study on Child Sexual Abuse in India: A Systematic Review (Choudhry et al., 2018) conducted a systematic review of the existing quantitative and qualitative studies on Child Sexual Abuse (CSA) and demonstrated that sexual violence is distributed among both boys and girls as well as this study demonstrates that the act of sexual abuse is an interplay between individual, familial and societal factors. Even though SDGs 5.1 and 5.2 talk explicitly about the violence perpetrated against women and SDG 16 talks about peaceful societies, the implication for the same for cis-men was not adequately mentioned.^3^ García-Moreno and Amin (2016) talk about the WHO’s detailed agenda for 2030 apropos any forms of violence (sexual or non-sexual) against women and girls which includes Mental health services for sexual violence survivors more importantly on providing appropriate care based on survey based research.

A study conducted by Walker (2020) on Harvard Medical Review mentions sexual abuse and violence against women as silent pandemic. The Safe City Programme in New Delhi was a program conducted by UN Women which is an organization dedicated to gender equality and empowerment of women as well as serves as the global champion for women and girls’ rights. As a part of this program, they conducted a survey on the high-and low-income areas of New Delhi, about 73 percent of the respondents reported that the city is unsafe as well as reported that women and men face sexual violence in their own neighborhoods. It foregrounds the lack of women’s spatial confidence in their own places. Rai and Rai’s (2020) study on Children and Youth Services Review explores how women’s confidence and fearlessness are undermined in their own city due to acts of sexual abuse and violence. It will be far worse for women and men when the act of sexual abuse is perpetrated by their own family clan and friends. All the participants in this study reported that they were sexually abused by people known to their families, friends, and relatives. Although much research has been done on sexual abuse crimes, and survivor’s narratives, there is a lack of research on the lived experiences of the survivors particularly on the southern part of India and ignorance on the part of the educators concerning this issue. So, this study proposes to work on the narrative of sexual abuse survivors who belongs to the community of the researchers.

## Method

### Participants

At the time of the study, the participants are in their first year of study at an engineering university and they are all female. Participants are addressed as Nithya, Geetha, Preetha, and Sheela to maintain anonymity (Original names changed). All the participants are from urban Chennai, and they were sexually abused during COVID-19 at the time of their late teen years.

### Data collection and procedure

This study begins by distributing a questionnaire to 500 participants to identify the participants who had experienced one or other forms of sexual abuse during COVID-19. The following questions were asked:

**Table 1:** 

S.No	Questions
1	Age?
2	Gender?
3	Type of Family?
4	Have you been asked to adjust to something you are not comfortable with to avoid family conflict?
5	Did you face any kind of sexual abuse during the pandemic? If yes, was it a one-time thing?
6	Did you find help to protect yourself?
7	Do you think COVID-19 paved the way for more sexual abuse cases?
8	Do you think, mobile phones are the main reason for sexual abuse during the COVID-19 pandemic?
9	Have you experienced any kind of sexual abuse through social media platforms like WhatsApp, Instagram, etc.?
10	Did you find protection after seeking help? If Yes, Whom did you first approach for help?
11	How well would you say you are educated about sex?
12	Do you think it is important to give sex education to children during their school days?
13	Do you think there is enough awareness about sexual abuse amongst older generations?
14	Are you willing to share? (In case we would like to know more about your trauma, will you be ready to share your experience to enhance the research outcomes and support policy making?) And, If you feel you were sexually abused/ are being abused, narrate the incident in a few words.

Only 258 participants responded to the questionnaire out of which 8 participants (including all genders) reported experiencing one or other forms of sexual abuse during COVID-19. Among the 8, four female participants are willing to share their experiences with the researchers. Secondly, brief interviews with the identified participants are conducted to record their lived experience of sexual abuse. Prior to the initiation of interviews, participants were reminded of the research’s objectives and furnished with detailed information via an information sheet. The voluntary nature of responding to interview questions was underscored, and participants were given the flexibility to withdraw at any point until one-month post-interview, leading to the removal of their interview record. Additionally, participants received a briefing on protocols safeguarding anonymity and confidentiality, including explicit clarification of scenarios where confidentiality might not be upheld (such as reporting information indicating a threat or risk to the participant or others or disclosing previously unknown offenses with identifiable victims). Informed consent was obtained through participants formally signing a consent form.

Semi-structured interviews were conducted, and their narratives were recorded using an Apple iPhone voice recorder. The interview schedule was decided based on the availability of the participants and the following unstructured questions were asked to obtain more information from the participants.

**Table 2:** 

S.No	Questions
1	Do you feel a change in the perception of your body after the incident?
2	Were you able to confront or continue your relationship with the perpetrator if the person is related to you?
3	What would have been done/ any prevention mechanisms or measures that you feel should be implemented to avoid these types of incidents?
4	Did you feel powerless because of the space?

Following the interview, the participants were given a chance to ask questions, and opportunities were given to them to add more. The recordings were transcribed using a transcription tool with potentially important information such as the name of the place, person, and other significant information removed.

### Phenomenology

Phenomenology is concerned with lived experience as a resource to foreground the underlying reality of the phenomenon that is being investigated. Merleau-Ponty et al.’s (2013) phenomenology emphasizes the notion of the body as a vehicle of our being in the world as a background or recessive presence. The shift in consciousness from a healthy state to a state of alienation from and objectification of the body in any physical act can be better understood through the first-hand account of lived experience. Its sole purpose is to clarify the meaning of the world. The main aim of Phenomenology is to explore how people make sense of what happens to them. It takes the participants’ articulation of their lived experience without any judgment and uses it as a source of knowledge for the future. It aims to bring about the biases, contradictions, and prejudices into light rather than experiences imposed with natural attitudes, errors, and biases. Any Phenomenological enquiry treats lived experience as data. More importantly, Interpretative (Interpretive) Phenomenological Analysis works on two layers: understanding the participant’s experience and how they make sense of their experience. There are three major Phenomenological traditions: Transcendental, Hermeneutic, and Existential.

Husserl (2001) said that the phenomenologist is “concerned with the essential structures of cognition and their essential correlation to things known” (p.27). Merleau-Ponty et al.’s (2013) states that phenomenologist attempts a “direct description of our experience as it is, without taking account of its psychological origin and the causal explanations, which the scientist, the historian or the sociologist may be able to provide” (p.6). Creswell et al.’s (2007) defines Phenomenology as a study that “describes the meaning for several individuals of their lived experiences of a concept or a phenomenon” (p. 62). The sole purpose of Phenomenology is to reorient the natural sciences perspective of treating the body as an exterior without an interior to a holistic understanding of subjectivity in meaning-making and understanding of the world Merleau-Ponty (1962). And Heidegger (2010) brings attention to the relationship between beings and their contexts.

Van Manen (2017) states all the phenomenological traditions intended for the bracketing of lived moment experience free from generalizations and preconceptions and he asserted that people could “reflect phenomenologically on the living meaning of the lived experience” (p. 813). Contemporary neuropsychology reiterates the postulates of memory studies in a different way. It states that memories are the phenomenology of retrospection but not an exact representation of the past. As far as temporality is concerned, any phenomenologist enquiry should consider the privileged position of the subject in terms of societal, cultural, political, and material presence. When it comes to applying phenomenological philosophy to empirical research, there were no clearly defined principles in the works of Husserl, Heidegger, and Marleau-Ponty but the confounding statement is that the elucidation of the meaning of an experience apropos to the subjectivity the researcher is exploring and the challenge lies in building a phenomenologically credible research.

### Interpretative phenomenological analysis

Interpretative phenomenological analysis (IPA) gained its popularity as a qualitative approach in the late twentieth century. It is more concerned with the interpretation of the lived experience both by the participant and the researcher. Husserl emphasized intentionality, which is how humans impose meaning on their experience. The earlier usage of IPA was focused on health psychology based on the interest of the pioneers, but later it was found to be a useful methodology for developing insights from individual experiences. Since this is idiographic, this methodology is useful for focusing on less data and in-depth analysis. Any successful IPA study would be useful for the detailed exploration of any phenomena highlighting new knowledge and understanding of the phenomena under study. Apart from its focus on individual experiences, IPA analysis can foreground the influence of the dominant social, cultural, and political factors on the interpretation (Lopez and Willis, 2004). And more importantly, this methodological approach focuses on the integrated self and demonstrates new understandings (Dickson et al., 2007).

Even though IPA offers a holistic understanding of the phenomenon under study, the researcher’s conception can affect and complicate the ‘process of interpretative activity’ (Smith, 1996). This ‘double hermeneutic’ (Smith et al., 1999) emphasizes the double interpretation of the experience: by the research participants and the researcher. An IPA study on male sexual offenders (Kloess et al., 2019) provided new insights into the diverse situational and vulnerability factors of the perpetrators in committing the act. A study on the survivor’s decision for legal initiation using Interpretative Phenomenological Analysis (Plastock et al., 2021) brings attention to the diverse factors that influence the survivor’s decision as to whether to initiate legal proceedings or not.

Similarly, this study employs a qualitative methodology using Interpretative Phenomenological Analysis, an idiographic approach whereby each experience is analyzed in detail apropos the given context (Smith, 2011). The first author herself interviewed the participants in both English and in the local language of Chennai (Tamil) and transcribed the recordings. Three participants did not express consent for recording, but they allowed the first author to record it in text. After each interview, the first author diligently noted her initial thoughts and notions in order to separate the preconceptions of the author from the intended focus/aim of the study (Tindall et al., 2009). Subsequently, the author analyzed the transcripts line by line to identify the factors affecting the act, experiential claims for the committed act, and the standpoint/positionality of the participants.

### Analysis

Four participants out of the 8 who experienced sexual abuse expressed willingness to narrate their experience with the researcher. Almost all the 8 participants believed that COVID-19 paved the way for more sexual abuse cases, and they stated that they were well aware of the helpline number for reporting child sexual abuse. This study aims to understand the experiences of the participants and how they perceive the experience (Make meaning of the experience). Three main themes emerged in this study: (a) *Material Circumstances as a cause of the incident (b) Patriarchal Indian family structure (c) Association of Shame*. ‘Material Circumstances as a Cause of the incident’ refers to how the material world including the material things, and the enclosed space influences the perpetrators to commit the act. ‘Patriarchal Indian family structure’ refers to how the Indian familial structure prevents them from disclosing it to their parents immediately. ‘Association of Shame’ refers to how associating shame with the incidents prevents the survivors from reporting the incident.

### Material circumstances as a cause of the incident

Among the selected participants, Sheela gave both a written narrative and recorded interview (personal) and this supplements her audio recording.

*‘It was one of my dad’s very very very close friend. We were almost like a family and I always imagined him as my father but during pandemic we spent most of the time together. Firstly, I stared to feel uncomfortable with his behavior, the way he talk and the intimate looks he gave me, then slowly he started to call me and talk rubbish about me in his life and then I told about this to friends and they always had my back and I recorded all the calls that he spoke to me. I was so afraid to say it to my parents because he was so close to my family. Later he started to abuse me physically whenever he comes to my home, once I got stuck with him in our home and he tried to miss behave with me. Later after few days we informed my parents, first when they knew they were bit angry on me since I did not tell them the first day itself but then they understood it, Then we almost thought of giving a police case on him but keeping my future in thoughts we just had some massive fights. But with god’s grace my family and friends had my back (Sheela).*

The experience of Sheela brings attention to the effect of the material world in the act of Sexual Abuse (SA). The perpetrator was a known friend to the family for more than 20 years and this relationship enabled easy access to the participant’s house. COVID-19 has imposed more restrictions on the mobility of people, therefore people often tend to visit relatives who are living nearby for recreational purposes. In the case of this participant, the perpetrator takes advantage of the fact of being a family friend staying next door as well as the COVID imposed restrictions in committing the act. As per the participant’s interpretation of the experience, the visit was more frequent during COVID-19 pandemic and the family had nowhere to go other than visiting nearby friends and relatives.

Initially, the survivor was experiencing inappropriate touch, but later during this time, the perpetrator took advantage of time and space and forcefully molested the survivor. This brings attention to the materiality/ material dimension of the experience. All 8 participants reported noticing a change in their close one’s behavior during the COVID-19 pandemic and it alerted us to the lack of mental health support and help during the crisis time. Similarly, in the case of Geetha, the perpetrator was the uncle who frequently visits the family, but the survivor reported the act before it got out of hand. At Sheela’s home, On the day of the incident.

‘*My mom and dad were not at home when he enters the house, and my sister was playing outside. He manipulated the timings and approached me. He forced me for a hug and asked me to kiss him and forcefully touching me inappropriately. I did not know how to react whether to shout or cry. I was confused, scared, and shocked’ (Sheela).*

She reported past incidents of inappropriate touching to her male friends. After she reported this incident, Whenever she was with her friends, they never let him near her. Apart from this incident, the perpetrator used to send obscene messages, videos, and photos to the survivor’s phone before the incident.

Nithya was not ready to disclose much due to her close affinity with the perpetrator. In her case, the perpetrator was the adopted brother, and it brings attention to the effect of spatiality and temporality in committing the act. Nithya’s parents adopted a male child before her birth, and they grew up in the same house as siblings. The survivor was always in close proximity, but the act was committed during the COVID-19 pandemic. Due to the imposed restrictions, people were forced to stay in their houses owing to the advantageous circumstances for the perpetrator to commit the crime. This act poses serious questions in relationship building, mental health, and stability in bringing up an adopted child along with the biological child.

Geetha was sexually abused by her uncle who often visits her home during the pandemic. This uncle often asks Geetha to sit on his lap and inappropriately touched her, so Geetha started to hide in her bathroom whenever the uncle visits their home. This brings attention to the lack of safe space for a girl in her own home. Prema have experienced sexual abuse during her commute to their tuition centers. The usually crowded autos were lacking people because of COVID-19, so the perpetrator used the situation to their own advantage and committed the act. The perpetrator inappropriately touched the girl, and he forced her to kiss his own private parts.

‘*He stopped the vehicle in an empty road and kissed me. He forced my face in to his penis and I was shocked, felt disgust and cried’ (Prema).*

Prema immediately reported the incident to her family and the auto men were not seen from the next day. One noticeable aspect mentioned by Geetha and Prema is they reported the incidents to the family without hesitation. Prema mentioned that she was able to report the incident because the perpetrator were unknown/not related to the family.

### Patriarchal Indian family structure

When the interviewer asked about reporting the incident to their parents, all the survivors reported that they were confused and not sure about their father’s reactions and felt scared to report the incidents.

*Interviewer: Did you feel like reporting this incident immediately to your parents?*

*Sheela: No. I was confused about my parents’ reaction towards this because this man is very close to my father, But I decided not to tell this to my father first because I felt my mom will understand it better than my father.*

After the participant’s family came to know about the incident, they decided to report it to the police, but they did not go for it considering its implications in the girl’s life as well as the perpetrator’s wife and children and Sheela has mentioned that she fought with her parents for not going to the police. This brings attention to the typical Indian familial setting and the patriarchal mindset that prevails in the families. In Indian families, any shame on girl children will be looked upon as a disgrace to the head of the family and household. Moreover, Indian parents associates womanhood with purity and they believe that exposing the incidents will reduce the chance of their daughters getting married.

‘A study on Childhood Attachments, sexual abuse, and their relationship to Adult Coping in Child Molesters’ (Marshall et al., 2000) demonstrated that the subjects of their study reported greater attachments to their mothers than fathers as well as explicated the correlation between the insecure patterns of childhood attachment and ineffective adult coping. All the participants feel that their mothers can understand and protect them than their fathers. A 2022 study on *Gender Norms, Domestic Violence, and the Southern Indian puzzle* (Chattopadhyay and Sidharth, 2022) *conducted* by FLAME University, Pune suggested that even though the macroeconomic changes were suggesting improvements, they did not lead to changes in gender norms. The reluctance of the daughters to report these abusive incidents to the father as well as their doubts in terms of acceptance sadly upholds the unchanged gender norms in Indian society.

*Interviewer: Why did you decide not to report this incident to the police?*

*Sheela: Because my parents were not ready to put me in trouble and we knew their family very well. One thing that concerns our parents are his kids and wife. So we did not go for it.*

Again, the same mindset prevents the parents from reporting the incident to the police or seeking legal help. Likewise, Geetha and Prema did not report this incident to the police, nor did they seek legal assistance. Again, in Geetha’s case, she felt safe talking about it to her mother rather than her father.

*Interviewer: Why did you decide to report this incident to your mother?*

*Geetha: Because I feel my mother understands me well and I doubt that my father might scold me so did not even think about that.*

Nithya’s experience was the result of Siblings Sexual Abuse (SSA). The social myth justifying the experimental sexual acts between brother and sister has persisted for a long time. Hardy (2001) argues that the victims of SSA exhibit the same psychological and emotional impact on girls as girls sexually abused by fathers. Carretier et al. (2022) in their study on the impact of sibling sexual abuse in adolescent girls highlights the importance of the need to treat SSA with special care. They highlighted that the survivor should be separated from the perpetrator in case of significant distress and threat to safety caused by the presence perpetrator. Even though Nithya experienced significant distress, signs of trauma, depression, and nervousness, she was not separated from her brother. They continue to live under the same roof. This brings attention to the patriarchal Indian family structure, lack of awareness, and ignorance on the aspect of creating safe spaces for the survivors.

### Association of shame

All the participants in this study reported that they immediately felt a sense of shame and shock after the incidents, and this prevented them from reporting it immediately to their parents and seeking help. According to a longitudinal study on the persistence of shame following sexual abuse (Feiring and Taska, 2005), the survivors reported feeling shame for a period of 6 years. Likewise, in this study the survivors reported shame after the act and felt guilty about disclosing it to their parents however Sheela reported the continuous inappropriate behavior of her family friend to his friends, and it foregrounds the safe space in relationships outside the family unit. Even though Geetha and Prema reported the incidents to their family, they said that they felt shame, disgust, and fear immediately after the incidents.

## Discussion

*How things shape the Mind?* (Malafouris, 2013) demonstrates the constitutive role played by the material world in a cognitive process. New Materialism suggests the ‘turn to matter’ focusing on the role of the material world drawing from philosophy, anthropology, and posthuman sociology (Braidotti, 2013). It assert that the world and historical developments arise from a diverse array of material forces spanning the physical, biological, psychological, social, and cultural realms (Barad, 1996). Posthuman thinkers like Braidotti (2013) emphasized the relational role played by the material world in the constitution of the self. Similarly, IPA focuses on the integrated self and the situated subjectivity through in-depth analysis of individual experiences. Sheela and Nithya’s narratives offer an enriched understanding of the associated materiality in the acts starting from mobile phones, sofas, and houses (enclosed spaces) as well as how the pandemic situation and its associated restrictions (social) were made advantageous by the perpetrators in committing the act. Sheela received obscene messages, verbal sexual abuse over the phone, and obscene pictures before the perpetrator actually committed the crime. Fear, confusion and shame have prevented the survivor from reporting the incidents of technological abuse. This incident suggests the able use of technology by the perpetrator but ineffective usage on the survivor’s part. It alerts us to the relationality of material things as well as their interactions with human beings in understanding any social problem.

Nithya was sexually abused by her adopted brother when they were sleeping on the bed next to each other as any siblings in a family. Her hesitation, self-doubt, and disgust after the incident explicate the lack of awareness, the society instilled fear and shame on females apropos body and sexuality. She cried during the interview and refused to talk further. Her difficulty in recollecting the abusive experience suggests an unspeakable and unresolved trauma in her life. She was referred to counseling by the interviewer (researcher) after the interview. After the incident, Nithya stayed in the same house with the perpetrator. She might have experienced severe trauma daily other than the time she was away from home. Now she is living in the college hostel far away from her home and the perpetrator got married as per the information given by her friend. Her refusal to talk about the actions on her parents’ part and the aftermath of the incident suggests a non-supportive response from her family. Geetha and Prema’s experiences are similar to the other girls in terms of known perpetrators, but the girls were more willing to talk like Sheela. Indian society’s family structure, parent’s fear about the future of girls (getting married), and the shame they felt on their part prevented them from reporting the incident or seeking legal help, but this societal and cultural influence left a traumatic scar on the girls’ lives. Sheela and Nithya reported feeling powerless during the incident and felt uncomfortable talking to or meeting (in a common gathering) the perpetrators. Above all, they emphasized on the educating and empowering the young girls to prevent sexual abuse and they stated this as a reason for talking about the incidents.

### Implications

According to a 1-year 2021 survey,^4^ the number of reported Sexual Abuse cases against women amounts to 12,500. The crimes committed by relatives, friends, or known people were higher compared to other reported crimes but still it is significantly underreported. According to the responses collected for this study, it is evident that almost all the participants who were sexually abused in one way or another do not want to talk about it. A 2023 study on *Ecological factors associated with sexual abuse among adolescent children in Mainland China* (Fu et al., 2023) suggested the involvement of parents, educational institutions, and government in comprehensive sex education among young children. We can adopt it for Indian schools and colleges. The first step against sexual abuse should be demonstrating the importance of disclosure like the *MeToo*^5^ movement in India. Secondly, creating awareness among young adults about acceptable and unacceptable sexual acts. Thirdly, the government bodies should ensure safety and enable easy access to report sexual abuse crimes. Then, the survivors should be encouraged to seek professional help in case of distress, and psychological trauma.

Few participants other than the selected participants for the study reported sexual abuse during their childhood and provided written narratives. It was observed that these participants were unaware of the difference between good touch and bad touch and this lack of awareness created an advantage for the perpetrator to sexually abuse them. All the participants who answered the questionnaire responded ‘YES’ to the importance of creating awareness about sex education among school children as well and 90 percent of the participants feel that there is not enough awareness of sexual abuse among older generations. As a part of this study, the researchers conducted an awareness campaign for the first-year students who participated in the study to create awareness of identifying potential perpetrators around them and discussed different avenues for reporting the incidents to seek help. This study was part of the lecture on contemporary issues for the corresponding semester. The second author (who is also a student) gave a talk on identifying potential perpetrators as well as discussed strategies to handle any potential risk situation followed by an interactive discussion. Students were actively participated in the study and gained confidence in talking about abusive incidents.

### Limitations

This study employed a qualitative approach to a small set of participants owing to the fact that this is a sensitive issue and people are finding it traumatic to talk about the same. The reluctance to disclose may be partly due to the stigmatization associated with it or the perpetrators are related to them either as family or friends, or they find it difficult to process the traumatic experience. Although the number of participants is perfectly justifiable for the methodology used, the reported themes and findings cannot be generalized across populations. The results are therefore not representative samples of sexual abuse survivors across the globe. Furthermore, the participants’ willingness to disclose the intricate details of their experiences varies, which affects the research outcomes.

## Conclusion

According to a 2021 study on the prevention of child sexual abuse (McCartan et al., 2021), professionals or practitioners all over the world agreed to the prevention of child sexual abuse as well as recognized that it is a public health issue. This study concluded that the professionals agreed that education is one of the best ways to work on the prevention of sexual abuse and the treatment of sexual abuse offenders but implied that it may not be applicable in all other countries due to the difference in social, cultural, and political background. Although this is starting to change with more studies/research taking place in different regions, there is still a lot more to be done. Studies focusing on cultivating resilience (Hinduja and Patchin, 2017) among the youth to resist and immediately report abusive behavior is one of the ways in which more offenses can be reported and survivors can seek help at an early stage. Three main themes emerged in this study which are the role played by the material things such as mobile phones, and houses, the material conditions that prevailed due to the COVID-19 outbreak, and the patriarchal family south Indian family structure. This study demonstrated how the material conditions that resulted as part of the COVID-19 pandemic became advantageous to the perpetrators as well as how the social, and cultural conditions were materialized in the act of sexual abuse.

## Data availability statement

The original contributions presented in the study are included in the article/supplementary material, further inquiries can be directed to the corresponding author.

## Ethics statement

The studies involving humans were approved by School of Social Sciences and Languages, VIT Chennai. The studies were conducted in accordance with the local legislation and institutional requirements. The participants provided their written informed consent to participate in this study. Written informed consent was obtained from the individual(s) for the publication of any potentially identifiable images or data included in this article.

## Author contributions

NG: Formal analysis, Methodology, Resources, Writing – original draft. BG: Resources, Writing – review & editing.

## Footnotes

^1^World Health Organization (WHO). Accessed February 1, 2024. https://www.who.int/home/search-results?indexCatalogue=genericsearchindex1&searchQuery=sexual%20abuse&wordsMode=AnyWord.

^2^“THE 17 GOALS.” Sustainable Development. Accessed February 1, 2024. https://sdgs.un.org/goals.

^3^Gender Equality and Women’s Empowerment.” United Nations Sustainable Development. Last modified September 7, 2023. https://www.un.org/sustainabledevelopment/gender-equality/.

^4^“India: Reported Crimes Against Women by Type 2021.” Statista. Last modified August 25, 2022. https://www.statista.com/statistics/633038/reported-crimes-against-women-by-type-india/.

^5^Metoo movement in India was started in 2018 by Tanushree Datta disclosing the sexual harassment of Padekar against her.
